# 5-Aminolevulinic acid improves chicken sperm motility

**DOI:** 10.5713/ab.21.0021

**Published:** 2021-04-23

**Authors:** Shin Taniguchi, Zhendong Zhu, Mei Matsuzaki, Masaoki Tsudzuki, Teruo Maeda

**Affiliations:** 1 Graduate School of Biosphere Science, Hiroshima University, Higashi-Hiroshima 739-8528, Japan; 2 One Health Business Department, Neopharma Japan Co., Ltd., Fujimi, Chiyodaku, Tokyo 102-0071, Japan; 3 Graduate School of Integrated Sciences for Life, Hiroshima University, Higashi-Hiroshima 739-8528, Japan; 4 Japanese Avian Bioresource Project Research Center, Hiroshima University, Higashi-Hiroshima 739-8528, Japan

**Keywords:** 5-Aminolevulinic Acid, Chicken, Sperm Motility

## Abstract

**Objective:**

This study investigated the effects of 5-aminolevulinic acid (5-ALA) on the motility parameters, mitochondrial membrane depolarization, and ATP levels in chicken sperm.

**Methods:**

The pooled semen from Barred Plymouth Rock males was used. In the first experiment, the semen was diluted 4-times with phosphate-buffered saline (PBS (−)) containing various concentrations (0, 0.01, 0.05, and 0.1 mM) of 5-ALA, and then the sperm motility parameters after incubation were evaluated by computer-assisted sperm analysis (CASA). In the second experiment, the semen was diluted 4-times with PBS (−) containing 0.05 mM 5-ALA, and then sperm mitochondrial membrane depolarization and ATP levels after 1.5 h of incubation were analyzed with the MitoPT® JC-1 Assay and ATP Assay kits, respectively. In the third experiment, the semen was removed from the seminal plasma and resuspended with the mediums of PBS (−), PBS (−) supplemented with CaCl_2_ and MgCl_2_ (PBS (+)) + 5-ALA, PBS (+) + caffeine, and PBS (+) + caffeine + 5-ALA. Then, the sperm motility parameters after incubation were evaluated by CASA. In the last experiment, the semen was treated with the mediums of PBS (−), PBS (−) + 5-ALA, 5.7% glucose, 5.7% glucose + 5-ALA after removing the seminal plasma, and then the sperm motility parameters were evaluated by CASA.

**Results:**

The addition of 0.05 mM 5-ALA significantly increased the chicken sperm motility, progressive motility, linearity, average path velocity, curvilinear velocity, straight-line velocity, and the wobble. The sperm mitochondrial membrane depolarization was also increased by the 5-ALA treatment. The 5-ALA treatment decreased the sperm ATP levels. Both the caffeine treatment and glucose treatment decreased the sperm motility during incubation period.

**Conclusion:**

5-ALA might increase sperm mitochondrial membrane depolarization to utilize the ATP for enhancing sperm movement.

## INTRODUCTION

5-Aminolevulinic acid (5-ALA) is a delta amino acid that has a carbonyl group at the fourth carbon [1], which naturally presents in plants, animals, algae, and photosynthetic bacteria [2–4]. There are two distinct pathways for 5-ALA to be biosynthesized; from glutamate in plants/bacteria or from the succinyl-coenzyme A and glycine in animals. 5-ALA is regarded as a precursor of heme, which is an essential substance for oxygen metabolism and energy generation in various animals [5]. Morokuma et al [6] reported that 5-ALA significantly enhanced hair growth and cell proliferation. Lee et al [7] demonstrated that the complex of 5-ALA and glycyl-histidyl-lysine peptide was considered as one of the complementary agents for the treatment of male-pattern hair loss. 5-ALA also has been reported to boost the immune system. Sato et al [8] reported that 5-ALA was used as an immunomodulator to stimulate T cells via mild oxidative stress in growing broiler chickens, thereby improving the growth performance. Moreover, when RAW264.7 cells were treated with a lipopolysaccharide stimulator, treatment with exogenous 5-ALA induced heme oxygenase-1 to downregulate the expression levels of nitric oxide and proinflammatory cytokines (tumor necrosis factor-α, cyclooxygenase 2, interleukin-1β, and interleukin-6), indicating that 5-ALA is an anti-inflammatory agent [9]. In recent years, 5-ALA has also been utilized in photodynamic therapy to diagnose brain tumors and determine the area of resection [10,11]. In addition, 5-ALA has been reported to be an important nutrient for plant growth [12,13]. Several studies have shown that 5-ALA can improve the salt tolerance of cotton seedlings [14] and cold resistance in rice seedlings [15]. However, to the best of our knowledge, there are still no reports on the effects of 5-ALA on animal reproduction systems. Whether 5-ALA is beneficial for improving the sperm quality remains unclear. Therefore, in the present study, we aimed to examine the effects of 5-ALA on the sperm motility parameters, mitochondrial membrane depolarization, and ATP levels in the chicken.

As the sperm motility patterns could be enhanced by some chemicals, such as caffeine and extracellular Ca^2+^ [16], we compared the role of caffeine, extracellular Ca^2+^ and 5-ALA on chicken sperm motility patterns to evaluate whether the mechanism of 5-ALA chemical enhancing sperm motility is similar to that mechanism in caffeine and extracellular Ca^2+^ or not. Moreover, to investigate whether 5-ALA improve chicken sperm motility by enhancing ATP production via glycolysis pathway or not, we compared the roles of 5-ALA in phosphate-buffered saline (PBS) (−) medium and the 5.7% glucose medium.

## MATERIALS AND METHODS

### Animals

Five Barred Plymouth Rock males (12 months old) were used. The males were individually caged and maintained in environmentally controlled houses on a 14 h light:10 h dark photoperiod. The feed and water were provided *ad libitum*. All of the animals used in the present study were handled in accordance with the regulations of the Animal Experiment Committee of Hiroshima University for animal experiments (approval number: C17–21).

### Semen collection

Semen samples were collected using the abdominal massage method of Burrows and Quinn [17]. Semen from five roosters was pooled to avoid the effects of individual males on the sperm motility.

### Experimental design

In the first experiment (Experiment 1), the fresh chicken semen was diluted with PBS (−); v:v = 1:3) containing various concentrations of 5-ALA (0, 0.01, 0.05, and 0.1 mM). The treated diluted semen was incubated at 37°C for 2 h. The sperm motility was evaluated every 30 min by computer-assisted sperm analysis (CASA).

To investigate how 5-ALA increase the sperm motility, we measured mitochondrial membrane depolarization and ATP levels of the sperm in the presence or absence of 0.05 mM 5-ALA after 1.5 h of incubation (Experiment 2).

In Experiment 3, semen was removed from the seminal plasma by washing two times and resuspended the sperm with mediums of PBS (−), PBS (−) supplemented with CaCl_2_ and MgCl_2_ (PBS (+)) + 5-ALA, PBS (+) + caffeine and PBS (+) + caffeine + 5-ALA. The sperm motility was evaluated at 15, 30, 60, and 90 min points of incubation by CASA. PBS (+) means the PBS medium contained Ca^2+^ and Mg^2+^ irons. The concentration of caffeine was 4 mM according to the report by Wishart and Ashizawa [16].

To further evaluate whether the 5-ALA enhanced sperm motility by utilizing ATP or generating ATP, we incubated the other sperm with mediums of PBS (−), PBS (−) + 5-ALA, glucose, glucose + 5-ALA for 1.5 h. The sperm motility was evaluated every 30 min by CASA (Experiment 4). The concertation of glucose was 5.7% (W/V), which is equilibrated with chicken seminal plasma [18].

### Evaluation of sperm motility parameters by computer-assisted sperm analysis system

According to Zhu et al [19], a total of 10 μL sample was placed in a pre-warmed counting chamber after incubation of sperm for different treatments. Sperm tracks (0.5 s, 45 frames) were captured at 60 Hz using a CASA system (HT CASA-Ceros II; Hamilton Thorne, Beverly, MA, USA). More than 200 individual trajectories were recorded in each treatment. The sperm parameters were defined as follows:

Motility (motile): Percentage of motile sperm moving with a path velocity >12 μm/s. Progress motility (progressive motile): Percentage of motile sperm moving with path velocity 45 μm/s and in a straight line for over 80% of the time. LIN, linearity; VAP, average path velocity; VCL, curvilinear velocity; VSL, straight-line velocity; WOB, wobble.

### Evaluation of sperm mitochondrial membrane depolarization

Sperm mitochondrial membrane depolarization was measured with MitoPT® JC-1 Assay (911, ImmunoChemistry Technologies, llc., Bloomington, MN, USA) according to Zhu et al [20]. Briefly, sperm samples after treatment were incubated with working solution for 5 min at 37°C in the dark. The mitochondrial membrane depolarization was analyzed by flow cytometry using a filter with a bandwidth of 574/26 nm (Attune R NxT Acoustic Focusing Cytometer, Invitrogen, Waltham, MA, USA). The mean fluorescence intensity (MFI) of JC-1 orange aggregates were measured. Sperm with high fluorescence intensity indicated sperm with high mitochondrial membrane depolarization. Total of 50,000 sperm events were analyzed.

### Measurement of sperm ATP levels

Intracellular ATP of sperm was measured as described previously by Matsuzaki et al [21]. The ejaculate was incubated with or without 0.05 mM 5-ALA for 1.5 h. The sperm was diluted 100-fold with PBS and dissolved in the ATP assay reagent (“Cellno” ATP Assay Reagent Ver.2, TOYO B-Net Co., Ltd., Tokyo, Japan). After incubating at room temperature for 10 min, the chemiluminescence signal was measured using a luminometer (ARVOTMX4, PerkinElmer Japan, Co., Ltd., Yokohama, Japan). Standard curves were prepared from ATP standard using serial dilutions to obtain concentrations of 0.75, 1.5, and 3 μM. The ATP levels in sperm samples were calculated according to the standard curve and expressed as pM/10^6^ of sperm.

### Statistical analysis

All the data were tested for normality and variance homogeneity prior to statistical analysis. The data were transformed by arcsine square root transformation when necessary, and analyzed using JMP 14.2 (SAS Institute Inc., Cary, NC, USA). The data from five replicates for comparison were performed by either two-way analysis of variance followed by Tukey-Kramer HDS test (motility parameters) or one-way analysis of variance followed by Student’s *t*-test (mitochondrial membrane depolarization and ATP levels). All the values from five replicates are presented as the mean±standard error. Treatments were considered statistically different from one another at p<0.05.

## RESULTS

Table 1 shows the effects of 5-ALA on sperm motility parameters during 2 h of incubation period (Experiment 1).

Supplementation with 0.01 and 0.05 mM 5-ALA significantly increased the sperm motility compared to the control group. However, the value of the motility in 0.1 mM 5-ALA treatment was similar to the control during the incubation period. Treatment with 0.05 mM 5-ALA showed the highest motility among all treatments.

The sperm progressive motility was significantly increased after 1 and 1.5 h of incubation. When compared to the control group, addition of 0.01 and 0.05 mM 5-ALA significantly increased the sperm progressive motility; moreover, the 0.05 mM 5-ALA treatment showed the highest value among all treatments. However, treatment with 0.1 mM 5-ALA did not improve the sperm progress motility.

The values of sperm LIN were improved after 1, 1.5, and 2 h of incubation. Treatment with 0.01 and 0.05 mM 5-ALA significantly improved the LIN compared to the control, whereas the 0.1 mM 5-ALA treatment did not significantly change compared to the control.

Sperm VAP increased during 1 to 2 h of incubation, and treatment with 0.05 mM 5-ALA significantly increased the VAP compared to the control at 0.5, 1, 1.5, and 2 h of incubation. The other treatments did not increase sperm VAP during the incubation period.

Treatment with 0.01 mM 5-ALA increased sperm VCL at 1 and 1.5 h of incubation, but no effect was observed at 2 h of incubation. Interestingly, compared to the control, treatment with 0.05 mM 5-ALA significantly increased sperm VCL at each point of incubation; however, the 0.1 mM 5-ALA treatment did not show any positive effect on the VCL parameter.

Regarding sperm VSL, the value of VSL was significantly increased at 1 and 1.5 h points of incubation. Treatment with 0.05 mM 5-ALA significantly increased the sperm VSL, whereas treatments with 0.01 and 0.1 mM 5-ALA did not improve sperm VSL.

As compared to the control, treatment with 0.01 mM 5-ALA improved the values of sperm WOB at 1 h of incubation, but no changes were observed at 1.5 h and 2 h of incubation. Interestingly, the sperm WOB was significantly increased when treated with 0.05 mM 5-ALA for 2 h. However, the sperm WOB was not increased in the 0.1 mM ALA treatment.

**Figure 1:**
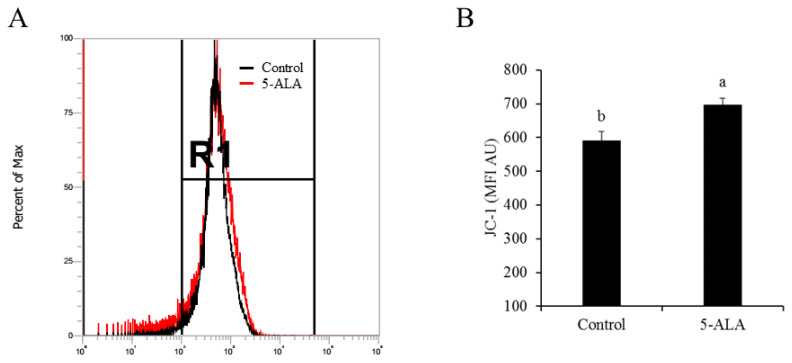
Effect of 5-aminolevulinic acid (5-ALA) treatment on sperm mitochondrial membrane depolarization. The sperm mitochondrial membrane depolarization after 1.5 h of incubation with 0.05 mM 5-ALA were analyzed with the MitoPT JC-1 Assay kit. (A) Fluorescence intensity of sperm when sperm analyzed by flow cytometry. Gate R1 means the chicken sperm positive stained with JC-1 probe. (B) Mean fluorescence intensity of sperm. Data were obtained from five replicates. ^a,b^ Different letters show significant difference (p<0.05).

**Figure 2:**
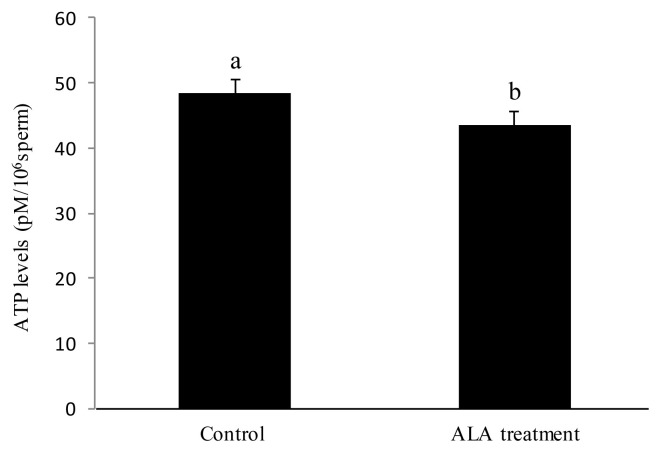
Effect of 5-aminolevulinic acid (5-ALA) treatment on sperm ATP levels. The sperm ATP levels after 1.5 h of incubation with 0.05 mM 5-ALA were analyzed with the ATP Assay kit. Data were obtained from five replicates. ^a,b^ Different letters show significant difference (p<0 .01).

As described above, sperm motility, progressive motility, LIN, VAP, VCL, VSL, and WOB were significantly increased by the 0.05 mM 5-ALA treatment after 1.5 h of incubation (Experiment 1); we also investigated whether the chicken sperm mitochondrial membrane depolarization was altered by 5-ALA. It was observed that addition of 5-ALA significantly increased the MFI of sperm when sperm were analyzed by flow cytometry (Figure 1A–B), which means that the sperm mitochondrial membrane depolarization was increased in the 0.05 mM ALA treatment as the high MFI indicates sperm with high mitochondrial membrane depolarization (Experiment 2). Interestingly, when we measured the sperm ATP level after ALA treatment (supplementation of 0.05 mM 5-ALA and 1.5 h incubation) with a ATP analyzed kit, it was observed that the ATP level in 5-ALA treatment was significantly (p<0.01) lower than the control (Figure 2) (Experiment 2).

**Figure 3:**
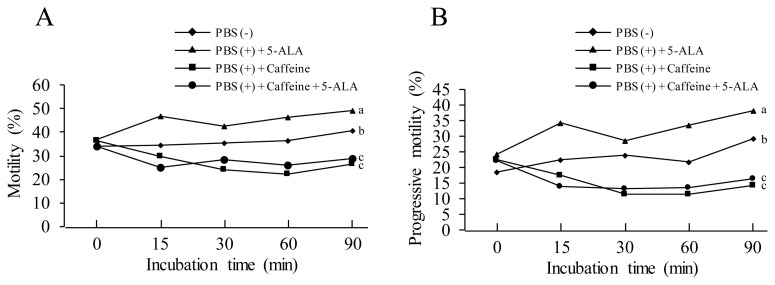
Effects of 5-aminolevulinic acid (5-ALA), caffeine and Ca^2+^ on sperm motility. The washed sperm was suspended with mediums of phosphate-buffered saline (PBS (−)), PBS (+) + 5-ALA, PBS (+) + caffeine and PBS (+) + caffeine + 5-ALA. The sperm motility was evaluated at 15, 30, 60, and 90 min points of incubation by computer-assisted sperm analysis. Data were obtained from five replicates. ^a–c^ Different letters show significant difference (p<0.05).

It was observed that PBS (+) + caffeine treatment significantly decreased sperm motility compared to the PBS (−) treatment, meanwhile, the PBS (+) + 5-ALA increased the motility during the incubation. Moreover, the positive effect of 5-ALA on sperm motility disappeared with the addition of the 5-ALA to the PBS (+) + caffeine treatment, which indicated that the 5-ALA could not rescue sperm motility in the treatment of PBS (+) + caffeine (Figure 3A). When we checked sperm progressive motility, the results were similar to those in motility pattern, PBS (+) + 5-ALA treatment showed the highest progressive motility among all treatments, and PBS (+) + caffeine decreased the sperm progressive motility. Addition of 5-ALA to the PBS (+) + caffeine treatment could not recover the sperm progressive motility (Figure 3B) (Experiment 3).

**Figure 4:**
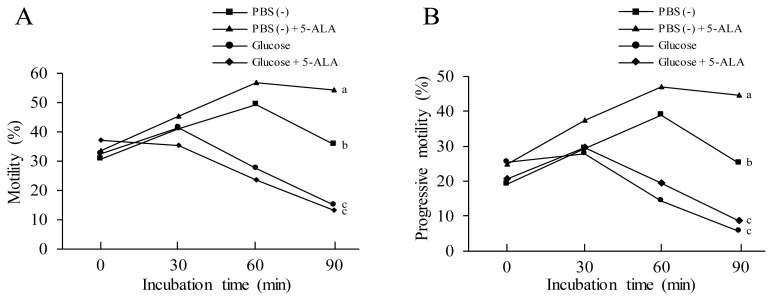
Effects of 5-aminolevulinic acid (5-ALA) and glucose on sperm motility. The washed sperm was incubated with mediums of phosphate-buffered saline (PBS (−)), PBS (−) + 5-ALA, glucose, glucose + 5-ALA for 1.5 h. The sperm motility was evaluated every 30 min by computer-assisted sperm analysis. Data were obtained from five replicates. ^a–c^ Different letters show significant difference (p<0.05).

As showed in Figure 4A, glucose medium decreased sperm motility compared to the PBS (−) treatment. Addition of 5-ALA to the PBS (−) medium significantly increased sperm motility, however, 5-ALA could not increase the motility in the glucose medium. Furthermore, the positive effect of 5-ALA on sperm progressive motility was observed in PBS (−) medium, but it did not increase sperm progressive motility in glucose medium (Figure 4B). Those data indicated that 5-ALA improved chicken motility patterns not rely on enhancing sperm ATP production via glycolysis metabolism (Experiment 4).

## DISCUSSION

To the best of our knowledge, this is the first time the effects of 5-ALA were evaluated in the animal reproduction field. It was observed that treatment with 5-ALA significantly increased chicken sperm motility, progressive motility, LIN, VAP, VCL, VSL, and WOB during the 2 h of incubation period in present study. Moreover, the sperm treated with 0.05 mM 5-ALA exhibited a high mitochondrial membrane depolarization. These results were consistent with those of previous studies, which reported that the mitochondrial membrane depolarization is highly positively correlated with sperm motility [20,22,23]. Mitochondria are one of major energy source generation sites in the sperm, and the sperm with high mitochondrial membrane depolarization indicates that they have high linear motility [19,20]. As the chicken sperm mitochondrial membrane depolarization was activated by 0.05 mM 5-ALA, the sperm was provided with amounts of energy for continuous movement, thus increasing the sperm motility patterns in this study. These data were similar to those of Chiabrando et al [5] who reported that 5-ALA is an essential substrate for enhancing the oxygen metabolism and energy generation in the animals. Moreover, Klimowicz-Bodys et al [24] found that sperm motility and progressive motility were also positively correlated with sperm viability in birds when the CASA was used to analyze sperm motility patterns, indicating that the viability of the chicken sperm in the present study was also improved by treatment with 5-ALA. The 5-ALA was beneficial for improving chicken sperm motility patterns during the incubation.

Chicken sperm mitochondrial membrane depolarization was enhanced by the 5-ALA treatment in the present study, which was consistent with the previous studies that the 5-ALA could stimulate the mitochondrial function in somatic cells [25,26]. Wachowska et al [27] demonstrated that the 5-ALA is an endogenous amino acid and an intermediate involved in heme biosynthesis in mitochondria. There are two steps involved in heme synthesis from 5-ALA: firstly, the endogenous or the exogenous 5-ALA is metabolized as a substrate for protoporphyrin IX, then the cell inserts a ferrous ion into protoporphyrin IX to generate heme. Moreover, heme acts as a protein-bound prosthetic group in the mitochondrial respiratory chain complexes II, III, and IV and cytochrome C [28], thus, the activity of the respiratory chain complexes is depended on the heme level, which indicates that the mitochondrial function is regulated by the heme. In present study, addition of 5-ALA significantly increased the sperm mitochondrial membrane depolarization (Figure 1A–B) maybe because the sperm utilizes 5-ALA to synthesise heme. It was supported by Shimura et al [29] who observed that 5-ALA upregulated oxidative phosphorylation proteins with increased oxidative phosphorylation gene expression in human skin fibroblasts. The result also agreed with the observation that 5-ALA was necessary to produce heme proteins [26,30–32]. Therefore, sperm may utilize the exogenous 5-ALA to produce heme to enhance mitochondrial function.

Sperm ATP is generated by the glycolysis and mitochondrial oxidative phosphorylation pathways [33]. In present study, it was observed that the ATP levels in the sperm were decreased by addition of 0.05 mM ALA to the diluted medium after 1.5 h of incubation. The results showed that the sperm ATP levels are negatively correlated with the data on linear motility patterns, which might be because the total levels of ATP rather than the mitochondria-generated ATP levels were measured in this study. Zhu et al [20] reported that only the ATPs generated from the mitochondrial oxidative phosphorylation are positively correlated with linear motility of the spermatozoa. Furthermore, when we added 5-ALA to the glucose medium, the sperm motility patterns were not increased (Figure 4A–B), which indicated that the 5-ALA could not enhance ATP production in sperm. Chicken sperm may utilize the 5-ALA for stimulating ATP utilization rather than ATP generation to increase sperm motility in this study. As sperm motility is correlated with both the ability to produce sufficient ATP and the ability to utilize ATP effectively [34], the ATP level in sperm will be decreased under the condition that sperm utilize the ATP for flagellum movement without enhancement of ATP generation. This supports our result in which a reduction of ATP levels was observed in the 5-ALA treatment in present study. Unfortunately, the mechanism of how 5-ALA stimulates chicken sperm utilization of ATP is not clear, and needs further study.

Regarding the regulation of the chicken sperm motility, Wishart and Ashizawa [16] reported that calcium and cAMP were the main regulators of sperm motility, and caffeine, but not calcium, caused an increase in sperm cAMP levels. In this study, the caffeine treatment could not recover the sperm motility (Figure 3A–B). Therefore, it is considered that 5-ALA and caffeine might be different functions to increase chicken sperm motility.

It was observed that the 0.05 mM is the best concentration of 5-ALA to improve the chicken sperm quality, while the high concentration (0.1 mM) had a negative effect on sperm motility in this study. The results were similar to the ones reported by Shimamura et al [35] who reported that high concentrations of 5-ALA resulted in a decrease in the cell viability. Previous studies showed that 5-ALA increased the reactive oxygen species (ROS) in the cells [35,36]; therefore, high level of 5-ALA might increase the chicken sperm ROS levels, and thus decreasing the sperm linear motility compared to the 0.05 mM 5-ALA treatment.

In conclusion, the present study is the first report on the effect of 5-ALA on sperm quality. We found that the supplementation with 0.05 mM 5-ALA significantly increased the chicken sperm motility, progressive motility, LIN, VAP, VCL, VSL, and the WOB. Moreover, sperm mitochondrial membrane depolarization was also increased by treatment with 0.05 mM 5-ALA. Interestingly, addition of 0.05 mM 5-ALA to the PBS medium decreased the sperm ATP levels after 1.5 h of incubation, whereas the sperm motility could not be increased by 0.05 mM 5-ALA treatment in the glucose medium. Therefore, 5-ALA might increase sperm mitochondrial membrane depolarization to utilize the ATP for enhancing sperm movement.

## Figures and Tables

**Table 1:** Effects of various concentrations of 5-aminolevulinic acid and the incubation time for sperm motility parameters

Item	Con (mM)	Time (h)				SEM	p-vale		

							Con^1)^	Time	Con×time
Motility^2)^ (%)		0.5^A^	1^A^	1.5^A^	2^A^				
						
	0^a^	62.1	65.3	65.0	58.0	4.3	0.0003	0.0348	0.6859
	0.01^bc^	64.9	69.2	66.8	66.6				
	0.05^c^	65.1	70.7	70.6	69.0				
	0.1^ab^	63.9	65.6	62.8	61.2				
Prog. M.^3)^ (%)		0.5^A^	1^B^	1.5^B^	2^AB^				
						
	0^a^	45.6	54.5	55.2	45.7	8.1	<0.0001	0.0068	0.8730
	0.01^bc^	52.6	59.9	57.4	56.1				
	0.05^c^	54.6	61.4	63.0	59.3				
	0.1^ab^	50.3	53.1	51.8	50.1				
LIN (%)		0.5^A^	1^B^	1.5^B^	2^B^				
						
	0^a^	48.0	54.9	56.2	54.3	1.7	<0.0001	<0.0001	0.2501
	0.01^bc^	51.3	58.8	59.9	56.0				
	0.05^c^	54.6	61.6	60.0	60.1				
	0.1^ab^	53.2	57.4	52.5	53.4				
VAP (mm)		0.5^A^	1^B^	1.5^B^	2^B^				
						
	0^a^	74.6	83.3	87.6	84.4	4.5	0.0166	0.0001	0.9969
	0.01^ab^	75.6	88.5	91.1	82.7				
	0.05^b^	83.0	94.5	97.1	90.9				
	0.1^ab^	75.2	89.0	86.6	84.0				
VCL (mm)		0.5^A^	1^B^	1.5^B^	2^AB^				
						
	0^a^	107.1	120.7	124.7	116.5	8.2	0.0063	0.0037	0.9914
	0.01^ab^	115.6	126.3	123.7	114.8				
	0.05^b^	119.6	134.8	135.1	125.6				
	0.1^a^	110.9	119.7	119.0	114.8				
VSL (mm)		0.5^A^	1^B^	1.5^B^	2^AB^				
						
	0^a^	92.1	109.2	109.8	105.1	19.8	0.0042	0.0004	0.9935
	0.01^ab^	99.9	115.2	113.2	102.6				
	0.05^b^	107.0	120.3	124.1	113.0				
	0.1^a^	95.3	109.8	105.8	101.0				
WOB (%)		0.5^A^	1^B^	1.5^AB^	2^AB^				
						
	0^a^	65.4	77.4	78.7	73.5	7.5	0.0009	0.0191	0.5568
	0.01^ab^	74.7	82.0	80.6	73.3				
	0.05^b^	76.3	86.5	83.4	81.1				
	0.1^a^	73.5	75.0	68.1	68.8				

Values are least square means.

The data obtained from five replicates were analyzed by two way ANOVA, and compared using Tukey-Kramer’s HDS test.

SEM, standard error of the mean; LIN, linearity; VAP, average path velocity; VCL, curvilinear velocity; VSL, straight-line velocity; WOB, wobble.

1) Con., concentration (mM).

2) Motility, percentage of motile sperm moving with path velocity >12 μm/s.

3) Prog. M., percentage of motile sperm moving with path velocity 45 μm/s and in a straight line over 80% of the time.

a–c Different letters within the same column are significantly different (p<0.05).

A,B Different letters within the same low are significantly different (p<0.05).
